# An Amharic Version of the Children’s Dermatology Life Quality Index (CDLQI): Cross‐Cultural Adaptation and Psychometric Evaluation

**DOI:** 10.1155/drp/4076478

**Published:** 2026-09-29

**Authors:** Debisa Eshatu Wendimu, Abebaw Yeshambel Alemu, Derese Bekele Daba, Saba Maria Lambert, Tedros Nigusse, Sagni Chali Jira, Yematawork Kebede Aragaw, Feleke Tilahun Zewdu, Wosen Ketema Bedaso, Fikregabrail Aberra Kassa, Demekech Damte, Teklu Cherkose, Amel Beshir Mohammed, Fewzia Shikur Mohammed, Shimelis Nigussie Doni, Michael Marks, Endalamaw Gadisa, Stephen L. Walker, Yohannes Hailemichael

**Affiliations:** ^1^ Malaria and Neglected Tropical Disease Division, Armauer Hansen Research Institute, Addis Ababa, Ethiopia, ahri.gov.et; ^2^ Department of Epidemiology and Biostatistics, College of Medicine and Health Sciences, Institute of Public Health, University of Gondar, Gondar, Ethiopia, uog.edu.et; ^3^ Department of Pediatric and Neonatal Nursing, College of Medicine and Health Sciences, Debre Tabor University, Debre Tabor, Ethiopia, dtu.edu.et; ^4^ All Africa Leprosy Tuberculosis and Rehabilitation Training Centre (ALERT) Comprehensive Specialized Hospital, Addis Ababa, Ethiopia; ^5^ Faculty of Infectious and Tropical Diseases, London School of Hygiene & Tropical Medicine, London, UK, lshtm.ac.uk; ^6^ Boru Meda General Hospital, Dessie, Ethiopia; ^7^ Department of Dermatovenereology, College of Health Sciences, Addis Ababa University, Addis Ababa, Ethiopia, aau.edu.et

**Keywords:** Amharic, children, Ethiopia, health related quality of life, reliability, validity

## Abstract

**Background:**

Skin diseases are associated with reduced health‐related quality of life (HRQoL) of children and their families. The Children’s Dermatology Life Quality Index (CDLQI) questionnaire is a skin disease‐specific measure of HRQoL in children. Our study aimed to translate the CDLQI into Amharic, culturally adapt, and evaluate the validity and reliability of the translation.

**Methods:**

This study was embedded within a larger hospital‐based cohort study of cutaneous leishmaniasis (CL). Forward–backward translation procedures were conducted, followed by assessment of content and face validity. The psychometric properties were evaluated through reliability (internal consistency and interrater reliability), construct validity (including known‐group, convergent, and discriminant validity), and responsiveness to change. Cronbach’s alpha, intraclass correlation coefficient (ICC), Mann–Whitney *U* test, Kruskal–Wallis test, confirmatory factor analysis (CFA), and Wilcoxon paired rank test were used to assess internal consistency, interrater reliability, known‐group validity, construct validity, and responsiveness to change validity, respectively.

**Results:**

The CDLQI showed acceptable content validity with a modified kappa between 0.7 and 0.9. In a pilot testing, children reported that the items in the translated Amharic version of the CDLQI were unambiguous. Among 188 enrolled participants, 129 (68.62%) were diagnosed with CL and 59 (31.38%) with other skin conditions. The median CDLQI score was 7 (IQR = 10). The overall Cronbach’s alpha and ICC were 0.82 and 0.92, respectively. The median CDLQI score showed statistically significant differences between “mild” vs. “severe” and “moderate” vs. “severe” CL cases (*p* < 0.001), as well as between the number of lesions (single vs. multiple) (*p* = 0.034) and lesion size (< 40 mm vs. ≥ 40 mm) (*p* < 0.001). CFA showed acceptable convergent validity with most items loading strongly (0.50–0.74), and Heterotrait–Monotrait ratio values ranging from 0.614 to 0.744. The CDLQI median score significantly decreased from Day 1 (median = 9) to Day 90 (median = 4; *p* < 0.001).

**Conclusions:**

Our Amharic version of the CDLQI showed acceptable validity and reliability, supporting its use in clinical practice and research to assess the HRQoL of Amharic‐speaking children with skin conditions in Ethiopia.

## 1. Introduction

Skin conditions are major contributors to global morbidity, collectively ranking as the fourth leading cause of nonfatal disease burden globally and among the top causes of years lived with disability across both high‐ and low‐income countries [1]. In southern Ethiopia one study reported 75% of schoolchildren had at least one skin disease, with the commonest being scabies, tungiasis, and dermatophyte fungal infections [2]. Skin conditions have social and psychological effects on children and their families and are associated with significant impairment of health‐related quality of life (HRQoL) [3–5].

Standardized measures of HRQoL are important tools to assess how skin conditions affect individuals and evaluate treatment effects [6]. General HRQoL assessment tools are less sensitive than skin‐specific tools to measure the direct impact of skin conditions [7]. The Children’s Dermatology Life Quality Index (CDLQI) is a brief, easy‐to‐administer, responsive, and reliable skin‐specific tool to assess the HRQoL of children aged four to 16 years with skin disease [8].

The CDLQI consists of 10 items which assess the effect of an individual’s skin disease in the week prior to the completion of the questionnaire. Each item is scored on a 4‐point scale from 0 to 3, giving a total score ranging from 0 to 30. Higher scores indicate worse quality of life. A score of 0–1 suggests no effect at all on the child’s life, 2–6 a small effect, 7–12 a moderate effect, 13–18 a very large effect, and 19–30 an extremely large effect on quality of life [9].

An Amharic version of DLQI for adults is available [10], but there was no Amharic version of the CDLQI. Amharic is the one of the official languages of Ethiopia and is spoken by more than 60 million people [11]. Translating the CDLQI into Amharic is important to ensure accurate and culturally relevant assessment of HRQoL among Amharic‐speaking Ethiopian children affected by skin conditions. An appropriate and validated translation enables researchers and clinicians to better assess the burden of dermatological diseases affecting children in Ethiopia. We aimed to translate the English version of the CDLQI into Amharic, culturally adapt, and evaluate the validity and reliability of the translated Amharic version.

## 2. Methods

### 2.1. Study Design and Setting

This was a longitudinal study conducted between February 2023 and September 2024 and partly nested in a cohort study of cutaneous leishmaniasis (CL) at the ALERT Comprehensive Specialized Hospital and Boru Meda General Hospital [12].

### 2.2. Translation and Cross‐Cultural Adaptation

#### 2.2.1. Translation

We followed the steps recommended for the cross‐cultural adaptation of research instruments [13]. The UK English version of the CDLQI [14] was translated into Amharic independently by two native Amharic speakers fluent in English. The two Amharic translations were then jointly reviewed by the translators, and a single Amharic version agreed. Back translation into English of the agreed Amharic version was then undertaken independently by two different native Amharic speakers who had not been involved in the original translations. The two back‐translated versions were jointly reviewed for similarity to the original English version.

#### 2.2.2. Cross‐Cultural Adaptation

The grammatical structure and clarity of questions and responses in the translated version were reviewed for similarity with the original English version and the back translation by a panel of five experts, consisting of three dermatovenerologists, a sociologist, and a public health expert.

#### 2.2.3. Content Validity

Seven professionals (a public health professional, two epidemiologists, two social scientists, and two senior dermatovenerologists) assessed the relevance of each item in the developed Amharic version of CDLQI using a four‐point Likert scale with the responses of “not relevant”, “somewhat relevant”, “quite relevant”, and “highly relevant”. Based on these ratings, we calculated the item‐level content validity index (I‐CVI) and the average scale‐level content validity index (S‐CVI/Ave). Moreover, we computed kappa statistics to account for the chance agreement. I‐CVI > 0.78, S‐CVI/Ave > 0.9, and modified kappa statistics > 0.4 were accepted [15].

#### 2.2.4. Face Validity

The prefinal Amharic version of the CDLQI was pilot tested with six children. We explored their understanding and the emotional impact of each item, as well as any unclear wording during interviews.

### 2.3. Validation and Reliability

#### 2.3.1. Study Participants

Children with confirmed CL or other skin diseases who were older than 4 years of age and less than 16 years old were eligible to participate. Children were diagnosed and recruited from the dermatology services of both hospitals by dermatologists. Children with CL, confirmed by slit‐skin smear microscopy, were classified based on clinical phenotype as having localized CL (LCL) or mucocutaneous CL (MCL) or diffuse CL (DCL) using study definitions [12].

#### 2.3.2. Sample Size and Data Collection

We wished to recruit 200 participants, giving more than 10 study participants per scale item [16]. The Amharic CDLQI version was administered to children by four trained data collectors. Sociodemographic and clinical data were collected on standard data collection forms.

#### 2.3.3. Reliability

To evaluate whether the Amharic version of the CDLQI was reproducible and consistent, we assessed the internal consistency and interrater reliability. To assess interrater reliability, two interviewers independently administered the tool to a subset of participants, with both interviews conducted on the same day at least 1 hour apart.

#### 2.3.4. Validity

We assessed the construct validity through known‐group, convergent, and discriminant validity analyses. Known‐group validity was evaluated by differentiating between individuals with CL categorized according to physician‐determined severity, CL lesion location (head/neck only, trunk and/or limbs only, and multiple body region), size of the largest CL lesion (less than 40 mm and 40 mm or larger), and CL clinical phenotypes (LCL and MCL) [12]. In addition, we examined the convergent validity (the degree to which items of a specific construct are highly correlated) and discriminant validity (constructs that are supposed to be unrelated are indeed distinct from each other).

#### 2.3.5. Responsiveness to Change

A subgroup of participants with CL completed the Amharic version of the CDLQI on a second occasion 90 days after the start of treatment. The treating physician reported their CL treatment outcomes as “worse”, “no change”, “minor improvement”, “substantial improvement”, and “cured” [12].

### 2.4. Data Management and Analysis

Anonymized data were entered into REDCap [17]. Data were analyzed using STATA Version 17 [18]. Descriptive statistics were summarized using mean, median, standard deviation (SD), and interquartile range (IQR). Cronbach’s alpha and intraclass correlation coefficient (ICC) using the two‐way random effect model with absolute agreement type were calculated to assess internal consistency and interrater reliability, respectively, with alpha values > 0.7 considered acceptable [16] and ICC values between 0.75–0.90 and > 0.90 indicating good and excellent reliability, respectively [19]. We used Mann–Whitney U and Kruskal–Wallis tests to assess the known‐group validity, with statistical significance set at a *p* value < 0.05. To assess convergent and discriminant validity, confirmatory factor analysis (CFA) was fitted based on the structural equation modeling (SEM) approach, and the fit indices were reported. Convergent validity was further examined using average variance extracted (AVE) and McDonald’s omega (*ω*) for multiitem domains. Discriminant validity was assessed using the Heterotrait–Monotrait ratio (HTMT) among domains with multiple indicators. We employed the Wilcoxon paired signed‐rank test to determine the significance of CDLQI score change between Day 1 and Day 90.

#### 2.4.1. Ethical Approval

Ethical approval was obtained from the AHRI/ALERT Ethics Review Committee (PO/23/21), the National Research Ethics Review Committee (7/2–506/m259/35), and the London School of Hygiene and Tropical Medicine (26,421). Permission was obtained from the copyright holder (Cardiff University) prior to undertaking the translation and adaptation of the CDLQI. Written, informed consent was obtained from an appropriate adult responsible for the participant. In addition, assent was obtained from study participants aged 12 to 16 years.

## 3. Results

### 3.1. Translation and Cultural Adaptation

The members of the committee reviewed the items and resolved contextual ambiguities before pretesting the prefinal Amharic version. The committee noted that some translations had been rendered in a yes/no response format that was not consistent with the original version. This was corrected following the committee’s review.

#### 3.1.1. Content Validity

All items except item 6 demonstrated good content validity (I‐CVI = 0.9–1). The modified kappa showed good to excellent agreement (0.7 to 0.9), and the overall S‐CVI was acceptable at 0.9 (Supporting file 1, Table S1). Item 6, which originally refers to “avoiding swimming or other sports”, had a slightly lower I‐CVI of 0.7, reflecting that some experts considered it less relevant in the Ethiopian context, where the proportion of individuals 15 years or older who report being able to swim is low [20]. To address this, the item was adapted by adding the word “activities” next to “other sports”, making it more culturally relevant while retaining the intent of the original item.

#### 3.1.2. Face Validity

The Amharic version of the CDLQI was pilot tested in six children (four boys and two girls) aged 10 to 14 years and they reported that the items were clear and the choice of responses are unambiguous.

### 3.2. Characteristics of Participants

One hundred and eighty‐eight participants were interviewed using the final Amharic CDLQI version (Supporting file 2), 129 (68.6%) were diagnosed with CL, and 59 (31.4%) had other skin conditions (Supporting file 3). The median age was 13 years (IQR = 5). For children with CL 87 (67.4%) had LCL, and 99 (76.7%) had lesions on the head and neck only (Table 1).

**TABLE 1:** Sociodemographic and clinical characteristics of participants.

Variable	Category	Number (%)	Median CDLQI score (IQR)
Age (mean = 12.13, SD = 3.66, median = 13, IQR = 5)	4–11 years	57 (30.3)	5.0 (7.0)
	12–16 years	131 (69.6)	8.0 (10.0)

Sex	Male	96 (51.0)	8.0 (10.0)
	Female	92 (48.9)	6.0 (9.5)

Educational status	No schooling	25 (13.3)	7.0 (10.0)
	Primary (Grade 1–8)	115 (61.1)	3.0 (6.0)
	Secondary (Grade 9–12)	45 (23.9)	8.0 (9.0)
	Post‐secondary (Diploma and above)	3 (1.7)	5.0 (9.0)

Diagnosis	CL	129 (68.6)	8.0 (10.0)
	Dermatitis	8 (4.3)	7.5 (11.0)
	Other inflammatory conditions	5 (2.7)	7.0 (1.0)
	Acne	13 (6.9)	6.0 (3.0)
	Superficial fungal infection	12 (6.4)	3.5 (12.5)
	Vitiligo	7 (3.7)	2.0 (2.0)
	Other skin conditions	14 (7.4)	3.0 (6.0)

Clinical phenotype of CL	LCL	87 (67.4)	8.00 (10.00)
	MCL	39 (30.2)	9.00 (11.00)
	DCL	3 (2.3)	14.00 (1.00)

Number of lesion	Single	77 (59.7)	6.00 (10.00)
	Multiple	52 (40.3)	10.50 (9.50)

Size of the largest lesion	< 40 mm	53 (41.1)	4.00 (7.00)
	≥ 40 mm	76 (58.9)	10.00 (10.00)

Site of the lesion	Head and neck only	99 (76.7)	8.00 (10.00)
	Trunk and/or limbs only	17 (13.2)	10.00 (9.00)
	Multiple body regions^∗^	13 (10.1)	10.00 (9.00)

Physician determined CL Severity	Mild	46 (35.7)	6.50 (8.00)
	Moderate	63 (48.8)	7.00 (10.00)
	Severe	20 (15.5)	13.50 (5.50)

^∗^Multiple body regions: lesions on both the head and neck region, and trunk and/or limbs region.

The median CDLQI score of all participants was seven (with a range of 0 to 27, IQR of 10) (Table 2). There was a significant difference between the CDLQI scores of individuals diagnosed with CL and those with other skin conditions (*p* = 0.004). The impact of skin conditions on children’s HRQoL was extremely large in 12 (6.4%) of participants, very large in 36 (19.2%), moderately large in 49 (26.1%), small in 67 (35.6%), and had no effect in 24 (12.8%).

**TABLE 2:** CDLQI scores at items and domain level (*n* = 188).

CDLQI	Min	Max	Median	IQR
Total Score	0	27	7	10
Symptoms and feelings domain	0	6	3	3
Item 1. Pain, itchy, scratching, and sore	0	3	1	1
Item 2. Worry or bothered, ashamed or became sad	0	3	1	2
Leisure domain	0	9	1	3
Item 4. Changing or wearing special clothes/shoes because of skin	0	3	0	2
Item 5. Going out, playing or doing hobbies	0	3	0	1
Item 6. Avoidance of swimming or other sports	0	3	0	1
School and holiday domain	0	3	0	2
Item 7. Problem with schooling and enjoying holidays	0	3	0	2
Personal relationships domain	0	6	1	2
Item 3. Friendships	0	3	0	1
Item 8. Teasing, bullying, avoiding by other people	0	3	1	2
Sleep domain	0	3	0	1
Item 9. Effect on sleep	0	3	0	1
Treatment domain	0	3	0	1
Item 10. Treatment problem	0	3	0	1

### 3.3. Reliability

#### 3.3.1. Internal Consistency

The overall 10‐item scale showed good internal consistency with Cronbach’s alpha of 0.82. All items contributed to the overall reliability of the tool, and none of the items improved reliability if removed (item deletion alpha ranging from 0.79 to 0.82) (Table 3).

**TABLE 3:** Cronbach’s alpha of the Amharic CDLQI if item removed.

Items	Cronbach’s alpha if item removed
Item 1. Symptom and feeling (pain, itchy, scratching, and sore)	0.82
Item 2. Symptoms and feelings (worry or bothered, ashamed or became sad)	0.80
Item 3. Personal relationships (friendships)	0.80
Item 4. Leisure (changing or wearing special clothes/shoes because of skin)	0.80
Item 5. Leisure (going out, playing or doing hobbies)	0.79
Item 6. Leisure (avoidance of swimming or other sports/running, jumping)	0.80
Item 7. Problem with schooling and enjoying holidays	0.80
Item 8. Personal relationships (teasing, bullying, avoiding by other people)	0.80
Item 9. Effect on sleep	0.80
Item 10. Treatment problem	0.81

#### 3.3.2. Interrater Reliability

Seventy‐two children having CL completed the Amharic CDLQI with two different data collectors. The ICC was 0.92 (95% CI: 0.88–0.95), indicating an excellent level of agreement between the two raters on the CDLQI scores. Moreover, the Bland–Altman plot (Figure 1) demonstrated that 69 of 72 (95.8%) individuals’ interobserver measurements were within acceptable limits of agreement (−4.24 to 4.46).

**FIGURE 1:**
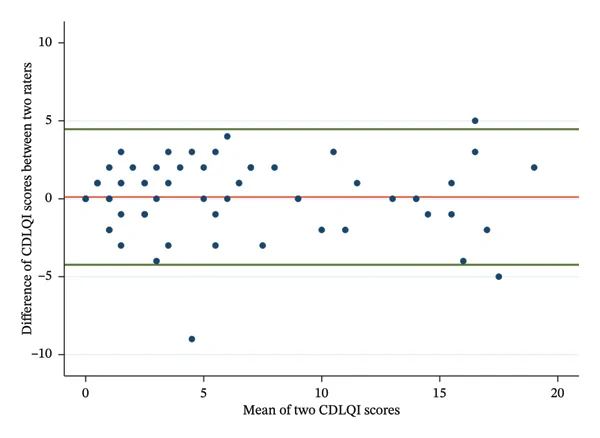
Bland–Altman plot of two interviewers applying the Amharic version of CDLQI (*n* = 72).

### 3.4. Validity

#### 3.4.1. Known Group Validity

The CDLQI score showed significant differences between individuals with CL categorized as “mild” or “severe” and those categorized as “moderate” or “severe”. Additionally, significant differences in CDLQI scores were observed between individuals with a single lesion and those with two or more, and lesions less than 40 mm compared to those with lesions 40 mm or larger (Table 4).

**TABLE 4:** Known group validity among CL patients between lesion site, CL severity, CL phenotype, number of lesion, and lesion size.

Variables (*n* = 129)	Overall *p* value (Kruskal–Wallis)	Effect size	Categories	*p* value (^∗^adjusted *p* value [bonferroni])
CL lesion sites	0.375	0.003	Head/neck vs. trunk/limbs	0.584
			Head/neck vs. multiple region	0.338
			Trunk/limbs vs. multiple region	0.999

Physician determined CL clinical severity	< 0.001	0.124	Mild vs. moderate	0.444
			Mild vs. severe	< 0.001^∗^
			Moderate vs. severe	0.001^∗^

CL phenotype	0.126	0.108	LCL vs. MCL	0.126

Number of lesion	0.034	0.028	Single vs multiple	0.034^∗^

CL lesion size	< 0.001	0.120	< 40 mm vs. ≥ 40 mm	< 0.001^∗^

^∗^Statistically significant at *p* value < 0.05.

#### 3.4.2. Convergent and Discriminant Validity

The CFA demonstrated acceptable convergent validity, with most items showing strong and statistically significant standardized loadings for multiitem domains (ranging between 0.50 and 0.74) above the acceptable threshold of 0.5. The AVE values were 0.40, 0.42, and 0.54 for domains 1, 2, and 4, respectively, while McDonald’s omega (*ω*) estimates were 0.549 for domain 1, 0.682 for 2, and 0.699 for 4 (Table 5).

**TABLE 5:** The standardized factor loadings of the 10‐item Amharic version CDLQI questionnaire across six factors (domains).

Factors (domains)	Item	Standardized factor loadings	Average variance extracted (AVE)	McDonald’s omega (*ω*)
Symptom and feeling	Item‐1	0.50	0.40	0.55
	Item‐2	0.73		

Leisure	Item‐4	0.53	0.42	0.68
	Item‐5	0.74		
	Item‐6	0.67		

Schooling	Item‐7	1.00	NA	NA

Relationship	Item‐3	0.74	0.54	0.70
	Item‐8	0.73		

Sleep	Item‐9	1.00	NA	NA

Treatment	Item‐10	1.00	NA	NA

*Note:* The model fit indices indicated overall model fit: root means square error of approximation (RMSEA) = 0.043 (< 0.08), standardized root means square residual (SRMR) = 0.036 (< 0.08), comparative fit index (CFI) = 0.982 (> 0.90), Tucker–Lewis Index (TLI) = 0.964 (> 0.90) and chi‐square *p* value = 0.127 (> 0.05), supporting the adequacy of the tool’s factor structure.

All estimated HTMT values were below the recommended threshold of 0.90, ranging from 0.614 to 0.744, suggesting adequate discriminant validity between the multiitem domains. Single‐item domains were not included in HTMT analysis because discriminant validity statistics require multiple indicators per latent construct (Table 6).

**TABLE 6:** Heterotrait–Monotrait ratio (HTMT) for discriminant validity of Amharic version CDLQI questionnaire.

Domains	Symptom and feeling	Leisure	Schooling	Relationship	Sleep	Treatment
Symptom and feeling	1.0					
Leisure	0.72	1.0				
Schooling	NA	NA	NA			
Relationship	0.61	0.74	NA	1.0		
Sleep	NA	NA	NA	NA	NA	NA
Treatment	NA	NA	NA	NA	NA	NA

#### 3.4.3. Responsiveness to Change

The responsiveness to change of the Amharic version of the CDLQI was evaluated in 96 children treated for CL, and the response was assessed by their physician at Day 90. The CDLQI median score at Day 90 was (median = 4, IQR = 6; *p* < 0.001) significantly lower than the score at Day 1 (median = 9, IQR = 9), *p* < 0.001, with a large effect size (*r* = 0.726). CDLQI scores at Day 90 were significantly lower compared to Day 1 for participants in whom the treating physician determined their outcome was ‘minor improvement’ or ‘substantial improvement’ or ‘cure’. There was no significant difference in CDLQI scores at Day 90 compared to pretreatment in those assessed by the physician as being worse or unchanged (Figure 2).

**FIGURE 2:**
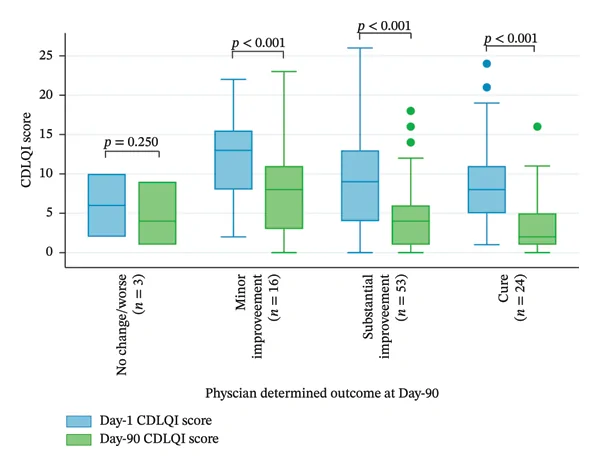
Boxplot of CDLQI total scores at Day 1 and Day 90, by physician determined outcome at Day 90.

## 4. Discussion

We translated and culturally adapted the CDLQI into Amharic by incorporating expert committee review and pilot testing with children to account for cultural differences, as described in other CDLQI adaptation studies [21, 22]. With regard to face validity, no child had difficulties in responding to the questions in the Amharic version of the CDLQI. The content validity indices were within the range, showing that the questionnaire items had high relevance and that the concept that it is intended to be measured was well covered.

The Amharic CDLQI demonstrated good internal consistency (*α* = 0.82) in with previous validation studies in Spanish, Turkish, and Cantonese versions of the CDLQI, where the range of Cronbach’s alpha reported was 0.82 to 0.92 [23–25]. This discrepancy may reflect differences in the study population, including variation in disease severity and clinical presentation, which can influence the variability and interitem correlations of quality‐of‐life responses. In addition, differences in the age distribution of participants may have contributed to the slightly lower internal consistency with other studies. The Amharic CDLQI demonstrated excellent interrater reliability (ICC = 0.92) and was higher than a previous study in children with localized scleroderma, where interrater reliability was rated good (ICC = 0.80) [26].

The Amharic CDLQI scores differed by disease severity, lesion number, and size in children diagnosed with CL. This aligns with studies from Turkey [24], Egypt [27], and Malaysia [28] among children with various skin conditions, where higher disease severity was consistently associated with greater HRQoL impairment. Our findings confirm the Amharic CDLQI can discriminate between groups with differing clinical severity. The CDLQI is sensitive to change, with scores decreasing significantly from baseline to Day 90 and reflecting physician‐rated clinical improvement. Other studies have reported similar findings [29–31], demonstrating the CDLQI’s ability to accurately represent clinical change and validating its utility for tracking treatment outcomes in pediatric dermatology.

The excellent CFA model fit and significant factor loadings support the convergent validity of the Amharic CDLQI. Although AVE and McDonald’s omega for the multiitem domains were below conventional thresholds, these findings should be interpreted cautiously because the original CDLQI includes several single‐item and two‐item domains, limiting the applicability of these indices. HTMT values below 0.85 supported adequate discriminant validity among the multiitem domains.

Our study has limitations. We did not compare our translated Amharic version of the CDLQI with an established measure of HRQoL. The majority of our participants had CL, and this may limit condition‐specific interpretation of the psychometric properties. The predominance of participants aged 12–16 years limits the generalizability of the findings to younger children aged 4–11 years. Future studies including a more balanced age distribution are warranted to further assess the instrument’s applicability and psychometric performance across the entire intended age range.

The validated Amharic CDLQI provides clinicians and researchers with a reliable tool to assess the psychosocial impact of dermatological conditions in Amharic‐speaking children. This can enhance routine clinical practice by informing treatment decisions and facilitating patient clinician communication. A validated skin‐specific Amharic PROM for children with skin disease is an essential research tool to assess the HRQoL of children with skin disease in Ethiopia and inform improved strategies for the management of dermatological conditions in children.

## Author Contributions

Conceptualization: Stephen L. Walker, Endalamaw Gadisa, Michael Marks, and Saba Maria Lambert.

Data Collection, management, and curation: Debisa Eshatu Wendimu, Derese Bekele Daba, Feleke Tilahun Zewdu, Amel Beshir Mohammed, Wosen Ketema Bedaso, Fewzia Shikur Mohammed, Shimelis Nigussie Doni, Yematawork Kebede Argaw, Demekech Damte, and Teklu Cherkose.

Formal analysis: Debisa Eshatu Wendimu and Abebaw Yeshambel Alemu.

Funding acquisition: Stephen L. Walker, Endalamaw Gadisa, and Michael Marks.

Project administration: Sagni Chali Jira and Saba Maria Lambert.

Software: Fikregabrail Aberra Kassa and Tedros Nigusse.

Supervision: Endalamaw Gadisa, Sagni Chali Jira, Yohannes Hailemichael, and Saba Maria Lambert.

Writing–original draft: Debisa Eshatu Wendimu and Abebaw Yeshambel Alemu.

Writing–review and editing: all authors.

## Funding

The Skin Health Africa Research Programme was funded by the Research on Interventions for Global Health Transformation Programme (Grant Reference Number NIHR200125) of the National Institute for Health and Care Research (NIHR) https://www.nihr.ac.uk/.

## Disclosure

The funder had no role in study design, data collection, and analysis.

## Ethics Statement

The study received ethical approval from the National Ethics Review Committee of Ethiopia (ref no: 7/2–506/m259/35), the ALERT/AHRI Ethical Review Committees (ref no: PO/23/21), and the London School of Hygiene and Tropical Medicine Ethics Committee (26,421).

## Conflicts of Interest

The authors declare no conflicts of interest.

## Supporting Information

Additional supporting information can be found online in the Supporting Information section.

## Supporting information


**Supporting Information 1** Supporting file 1: Content validity result table.

**Supporting Information 2** Supporting file 2: Final Amharic version of CDLQI.

**Supporting Information 3** Supporting file 3: Summary of skin conditions table.

## Data Availability

The data that support the findings of this study are available from the corresponding author upon reasonable request.
