# Supplementary material for: An Amharic Version of the Children’s Dermatology Life Quality Index (CDLQI): Cross‐Cultural Adaptation and Psychometric Evaluation
Source: Dermatol Res Pract. 2026 Sep 29;2026:4076478. doi: 10.1155/drp/4076478 (PMC13624295; doi:10.1155/drp/4076478)
Supplement: Supplementary file 1 [file DRP-2026-4076478-s001.docx]

**Appendix 1. Summary of Skin Conditions**

| **Category** | **Number of Cases (%)** |
| --- | --- |
| CL | 129 (68.6) |
| Dermatitis | 8 (4.2) |
| Acne | 13 (6.9) |
| Vitiligo | 7 (3.7) |
| **Other Skin Conditions** | |
| Pityriasis alba | 4 (2.1) |
| Varicella Zoster | 1 (0.5) |
| Pityriasis rosea | 2 (1.1) |
| Lymphangioma circumscriptum | 1 (0.5) |
| Wart | 2 (1.1) |
| Toxic Epidermal Necrolysis | 1 (0.5) |
| Mycetoma | 1 (0.5) |
| Molluscum contagiosum | 1 (0.5) |
| Keloid scar | 1 (0.5) |
| Keratosis pilaris | 1 (0.5) |
| **Other Inflammatory Skin Diseases** | |
| Psoriasis | 1 (0.5) |
| Lichen planus | 2 (1.1) |
| Folliculitis | 1 (0.5) |
| Lichen simplex chronicus | 1 (0.5) |
| **Superficial Fungal Infections** | |
| Tinea corporis | 4 (2.1) |
| Onychomycosis | 5 (2.7) |
| Tinea capitis | 3 (1.6) |
