# Supplementary material for: An Amharic Version of the Children’s Dermatology Life Quality Index (CDLQI): Cross‐Cultural Adaptation and Psychometric Evaluation
Source: Dermatol Res Pract. 2026 Sep 29;2026:4076478. doi: 10.1155/drp/4076478 (PMC13624295; doi:10.1155/drp/4076478)
Supplement: Supplementary file 2 [file DRP-2026-4076478-s002.docx]

Table 1. Content validity of CDLQI based on seven experts’ evaluation for the relevance of each item

|  | Description of item | Agreement | I-CVI | P_c_ | Modified kappa | Evaluation |
| --- | --- | --- | --- | --- | --- | --- |
| Item 1 | Over the last week, on your body part where you have the skin lesion, how painful, itchy and sore had it been? | 7 | 1.0 | 0.008 | 1.0 | Excellent |
| Item 2 | Over the last week, how much did you worry, bothered, ashamed or become sad about your skin lesion | 7 | 1.0 | 0.008 | 1.0 | Excellent |
| Item 3 | Over the last week, how much has your skin affected your friendships? | 6 | 0.9 | 0.050 | 0.8 | Excellent |
| Item 4 | Over the last week, how much have you worn or changed special clothes/shoes because of skin problems? | 6 | 0.9 | 0.050 | 0.8 | Excellent |
| Item 5 | Over the last week, how much has your skin trouble affected going out, playing, or doing hobbies? | 7 | 1.0 | 0.008 | 1.0 | Excellent |
| Item 6 | Over the last week, how much have you avoided swimming or other sport activities? | 5 | 0.7 | 0.164 | 0.7 | Good |
| Item 7 | Over the last week, were you having schooling time or a holiday?   a. If school time: Over the last week, how much did your skin affect your school work? b. If holiday time: How much over the last week, has your skin problem interfered with your enjoyment of the holiday? | 7 | 1.0 | 0.008 | 1.0 | Excellent |
| Item 8 | Over the last week, how much trouble have you had because of your skin with  other people calling you names, teasing, bullying, asking questions or avoiding you? | 7 | 1.0 | 0.008 | 1.0 | Excellent |
| Item 9 | Over the last week. how much has your sleep been affected by your skin problem? | 6 | 0.9 | 0.050 | 0.8 | Excellent |
| Item 10 | Over the last week, how much of a problem has the treatment for your skin been? | 7 | 1.0 | 0.008 | 1.0 | Excellent |
|  | **S-CVI/Ave=0.9** | | | | | |
