# Supplementary material for: An Amharic Version of the Children’s Dermatology Life Quality Index (CDLQI): Cross‐Cultural Adaptation and Psychometric Evaluation
Source: Dermatol Res Pract. 2026 Sep 29;2026:4076478. doi: 10.1155/drp/4076478 (PMC13624295; doi:10.1155/drp/4076478)
Supplement: Supplementary file 3 [file DRP-2026-4076478-s003.docx]

**CHILDREN'S DERMATOLOGY LIFE QUALITY INDEX**

**የሆስፒታል ቁጥር­­­­­­­ ___________________________**

**ስም:­­­­­­­­­-__________________________________________________________________	­­­**

**ምርመራ/የህመሙ አይነት:________________________________________________	CDLQI**

**ዕድሜ:	________________________________________________________	SCORE:**

**አድራሻ:_______________________________________________**

**ፆታ**

**ቁስሉ ያለበት ቦታ__________________________________________**

**ቀን:	_______________________________________________**

**ቃለ ምልልሱን ያደረገው ሰው ስም ________________________________________________________________**

የዚህ መጠይቅ ዓላማ የቆዳህ ሁኔታ በአጠቃላይ በህይወትህ/ሸ ላይ ባለፈው ሳምንት ያሳደረውን ተፅዕኖ ለመገምገም ነው፡፡ እባክህን/ሽን ለእያንዳንዱ ጥያቄ ተገቢውን መልስ ስጥ/ስጪ

| 1 | ባለፈው ሳምንት የቆዳ ህመም ያለበት ቦታ ላይ ምን ያህል የማሳከክ፣ የቁስለት ወይም የህመም ስሜት ነበረው? | እጅግ በጣም  በጣም  በመጠኑ  አልነበረውም | □  □  □  □ |
| --- | --- | --- | --- |
| 2 | ባለፈው ሳምንት የቆዳ በሽታህ/ሽ ምን ያሕል አሳፍሮሽ ወይም አሳስቦህ፣ አበሳጭቶህ/ሽ ወይም አሳዝኖህ/ሸ ነበር? | እጅግ በጣም  በጣም  በመጠኑ  አልነበረውም | □  □  □  □ |
| 3 | ባለፈው ሳምንት የቆዳህ/ሽ በሽታ ከጓኞችህ/ሸ በነበረህ/ሽ ግኑኝነት ላይ ምን ያህል ችግር ፈጥሮብሃል/ሻል? | እጅግ በጣም  በጣም  በመጠኑ  አልነበረውም | □  □  □  □ |
| 4 | ባለፈው ሳምንት በቆዳ በሽታ ምክንያት ምን ያህል ልብህን/ሽን ቀይረሀል/ሻል ወይም የተለየልብስ/ጫማ ለብሰሀል/ሻል? | እጅግ በጣም  በጣም  በመጠኑ  አልነበረውም | □  □  □  □ |
| 5 | ባለፈው ሳምንት በቆዳ ህመምህ/ሽ የምታደርገ/ጊውን እንቅስቃሴ (ከቤት ውጭ መላላክ)፤ መዝናናት (ጨዋታ)፣ ወደህ/ሽ የምታደርጋቸው ድርጊቶች ላይ ምንያህል ተጽዕኖ ፈጥሮብሃል/ሻል? | እጅግ በጣም  በጣም  በመጠኑ  አልነበረውም | □  □  □  □ |
| 6 | ባለፈው ሳምንት በቆዳ ሁኔታህ/ሽ ከዉሃ ዋና እና ሌሎች የስፖርት እንቅስቃሴዎችን እንዳታደርግ/ጊ ምን ያሕል ተጽዕኖ ፈጥሮብሃል/ሻል? | እጅግ በጣም  በጣም  በመጠኑ  አልነበረውም | □  □  □  □ |
| 7 | ያለፈው ሳምንት |  |  |
|  | የትምህርት ጊዜ ወይስ የረፍት ግዜ ነበረ  □ የትምህርት ግዜ ከነበረ  የቆዳህ/ሽ ሁኔታህ ትምህርትህ/ሽ (ትመህርት መከታተል፡ የቤት ስራ መስራት፡ ማጥናት) ላይ ምን ያህል ተጽኖ  ነበረው? | እጅግ በጣም  በጣም  በመጠኑ  አልነበረውም | □  □  □  □ |
|  | □ የእረፈት ሳምንት ከነበረ የቆዳህ/ሽ  ሁኔታህ እረፍትህን/ሽን በጥሩ ሁኔታ በማሳለፍ ላይ ምን ያህል ተጽኖ  ነበረው? | እጅግ በጣም  በጣም  በመጠኑ  አልነበረውም | □  □  □  □ |
| 8 | ባለፈው ሳምንት ውስጥ የቆዳ ሁኔታህ/ሽ ከሌሎች ሰዎች ያለህን/ሽን ግንኙነትን ላይ ምን ያህል ተጽኖ ነበረው፤ በስም በመጥራት፣ በመቀለድ፣ማስፈራራት፣ ጥያቄ በመጠየቅ ወይም ማግለል? | እጅግ በጣም  በጣም  በመጠኑ  አልነበረውም | □  □  □  □ |
| 9 | ባለፈው ሳምንት የቆዳህ/ሽ ሁኔታ ምን ያህል የእንቅልፍ ማጣት ችግር አስከትሎብሀል/ሻል | እጅግ በጣም  በጣም  በመጠኑ  አልነበረውም | □  □  □  □ |
| 10 | ባለፈው ሳምንት ለቆዳ ህመም የወሰደከው/ሽው መድሀኒት ያደረሰብህ/ሽ ችግር ምን  ያህል ነበር? | እጅግ በጣም  በጣም  በመጠኑ  አልነበረውም | □  □  □  □ |

እባክዎን ሁሉንም ጥያቄዎች መመለስዎን ያረጋግጡ እናመሰግናለን:

M.S. Lewis-Jones, A.Y. Finlay, May 1993, ከአሳታሚ ዎቹ ፍቃድ ውጪ ማባዛት የተከለከለነው፡፡
